
JOURNAL INFORMATION
==============================
NLM Title Abbreviation: ScientificWorldJournal
Journal ID: ScientificWorldJournal
NLM Journal ID: 101131163
Journal ID: TSWJ

The Scientific World Journal

ISSN: 2356-6140
EISSN: 1537-744X
Publisher: Wiley

ARTICLE INFORMATION
==============================
PMCID: PMC6009357
PMID: 29973793
DOI: 10.1100/tsw.2002.19
Article ID: 289427
Article version: 1
Subjects: Short Report

Extraction and Purification of Proteins from E. Coli without Harvesting Cells

Grabski Anthony *
Drott Don
Handley Mark
Mehler Mark
Novy Robert
Novagen, Inc., 601 Science Drive, Madison, WI 53711, USA
*Anthony Grabski: tony.grabski@novagen.com
Publication date: 2002
Electronic publication date: 2002 Jan 23
Volume: 2
First page: 36
Last page: 38
Copyright: Copyright © 2002 Anthony Grabski et al.
Copyright year: 2002
License: This is an open access article distributed under the Creative Commons Attribution License, which permits unrestricted use, distribution, and reproduction in any medium, provided the original work is properly cited.
License URL: https://creativecommons.org/licenses/by/3.0/

==============================
Advances in Gene Technology: The Genome and Beyond –
Structural Biology for Medicine (Proceedings of the 2002 Miami
*Nature Biotechnology* Winter Symposium)
*TheScientificWorld* 2002, 2(S2), 36–38
ISSN 1532-2246; DOI 10.1100/tsw.2002.19

### EXTRACTION AND PURIFICATION OF PROTEINS FROM *E. coli* WITHOUT HARVESTING CELLS

Anthony Grabski, Don Drott, Mark Handley, Mark Mehler, and Robert Novy
Novagen, Inc., 601 Science Drive, Madison, WI 53711
tony.grabski@novagen.com

**INTRODUCTION.** Extraction and separation of foreign proteins expressed in *E. coli* commonly employs multistep fractionation procedures combining centrifugation, mechanical disruption, volume reduction, filtration, and finally capture and recovery in order to separate cells from culture media and purify the target protein from the cell extracts[1,2]. These methods are not amenable to rapid high throughput (HT) purification of thousands of proteins. We demonstrate here a HT-compatible reagent, PopCulture™, and associated method that eliminate the necessity for multiple mechanical fractionation procedures. The entire culturing, extraction, and purification can be achieved in the original culture tube or multiwell plate. This "in-media" protein purification procedure may be adapted to high throughput robotic processing of samples for proteomics research and any application that would benefit from the increased speed and convenience it provides.

**METHOD.** Protein is isolated by culturing cells in liquid media under conditions for optimal target protein expression, chemically lysing the cells directly in their liquid media with a concentrated mixture of specialized detergents and buffer (*PopCulture™ Reagent), reduction of the extract's viscosity by the addition of *Benzonase® nuclease and *lysozyme, capturing the target protein by adsorption to an *affinity matrix, washing the capture matrix-target protein complex to remove spent culture media and unwanted cellular contaminants, and elution of the purified protein from the adsorptive matrix (*Novagen Inc., Madison, WI).

**RESULTS.** We have employed the PopCulture Reagent and protocol to purify a GST Tag™/His Tag® fusion protein using immobilized glutathione (GST-Bind Resins) or immobilized metal chelation (IMAC; His-Bind® Resins). The results are summarized in Table 1 and Figs. 1 and 2.

**Table I. Purification of His•Tag® GST expressed in *E. coli***

| Purification Method | Yield[1] | Purity[2] |
|---|---|---|
| Standard His•Bind® | 74 | 83 |
| PopCulture™ His•Bind | 111 | 89 |
| PopCulture Ni-NTA His•Bind | 170 | 85 |
| PopCulture His•Bind Magnetic[3] | 128 | 94 |
| Standard GST•Bind™ | 42 | 92 |
| PopCulture GST•Bind | 45 | 90 |
| PopCulture GST•Bind Magnetic[3] | 40 | 94 |

1. Yield in micrograms of target protein purified per ml of culture, as determined by BCA protein assay.
2. % purity determined by scanning densitometry of Coomassie blue stained SDS polyacrylamide gels.
3. Data represent the average of 8 separate wells processed in parallel.

**Figure 1. IMAC purification of a His•Tag fusion protein from *E. coli* total culture extracts**

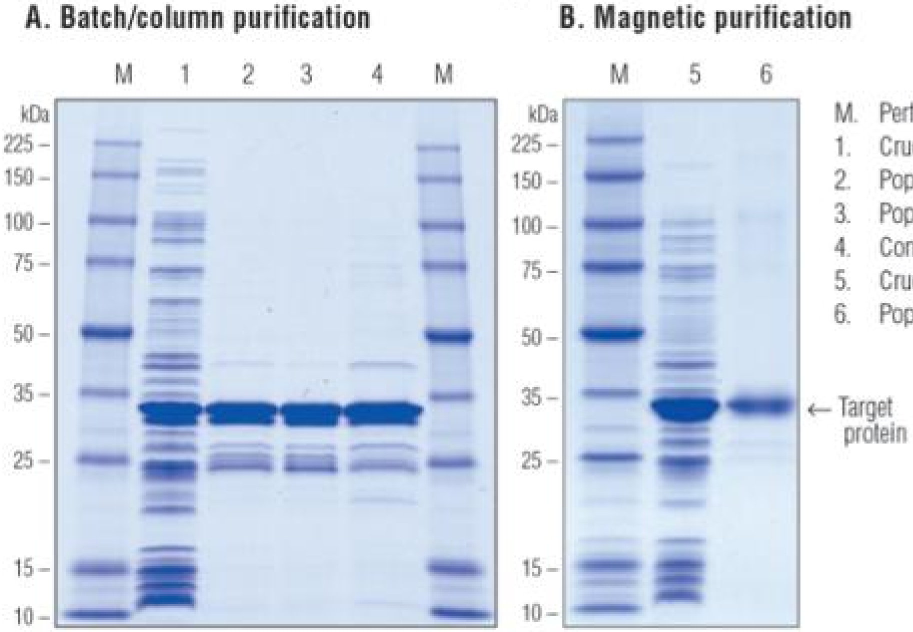

M. Perfect Protein™ Markers (10–225 kDa)
1. Crude extract
2. PopCulture + His•Bind Resin
3. PopCulture + Ni-NTA His•Bind Resin
4. Control + His•Bind Resin
5. Crude extract
6. PopCulture + His•Bind Mag. Agarose

**Figure 2. GST•Bind purification of a GST fusion protein from *E. coli* total culture extracts**

**A. Batch/column purification** **B. Magnetic purification**

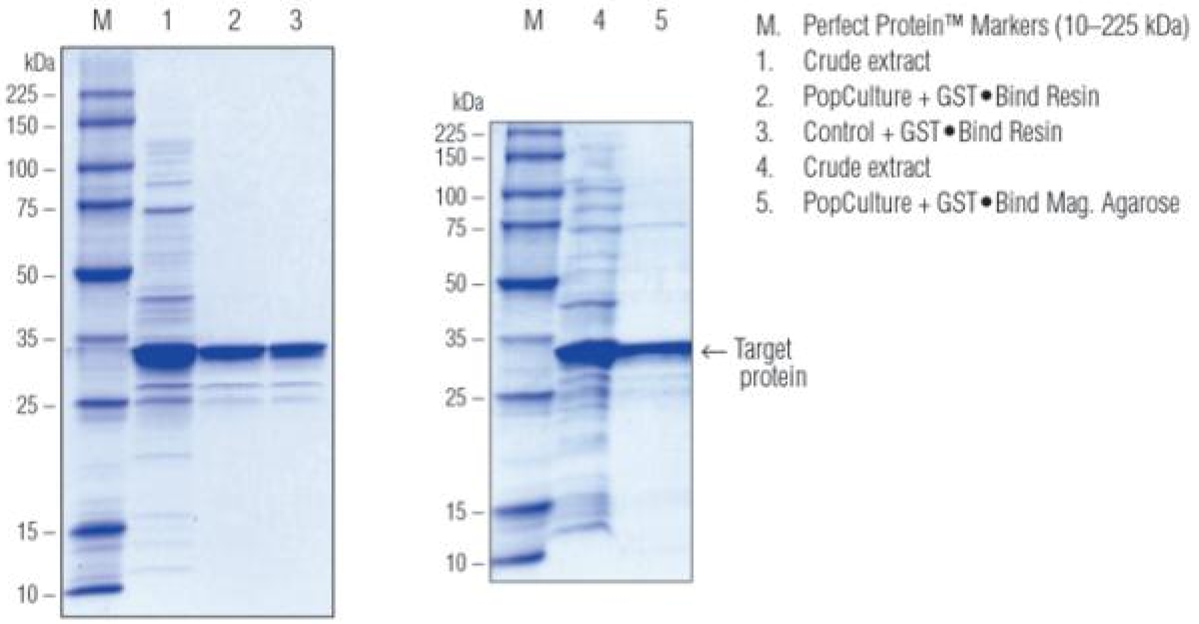

**DISCUSSION.** Extraction and purification of *E. coli* expressed proteins has been simplified through the application of specialized reagents that eliminate the need for mechanical disruption of cells[3]. This led us to consider the possibility that a concentrated formulation of detergents could achieve similar performance when added directly to unprocessed cultures, eliminating the need to collect the cells. The examples here demonstrate that direct lysis and affinity purification of total culture lysates can produce high yields of target proteins with purity and quality similar to conventionally purified preparations.

**REFERENCES**

1. Burgess, R.R., Ed. (1987) Protein Purification: Micro to Macro. Alan R. Liss, New York.
2. Deutscher, M.P., Ed. (1990) Methods in Enzymology 182. Guide to Protein Purification. Academic Press, New York.
3. Grabski, A.C., McCormick, M., and Mierendorf, R. (1999) in**Nova**tions 10, 17–19.
